# Altered core depressive symptom balance in post-COVID fatigue: reduced depressed mood in an exploratory matched historical-control study

**DOI:** 10.3389/fpsyt.2026.1877162

**Published:** 2026-08-04

**Authors:** Massimo Tusconi, Michele Fornaro, Michela Atzeni, Elisa Cantone, Elisa Pintus, Diego Primavera, Antonio E. Nardi, Mauro Giovanni Carta

**Affiliations:** 1 Center of Liaison Psychiatry and Psychosomatics, University Hospital of Cagliari, Cagliari, Italy; 2 Department of Neuroscience, Reproductive Science and Odontostomatology, Federico II University of Naples, Naples, Italy; 3 Department of Medicine and Surgery, University of Kore, Enna, EN, Italy; 4 Department of Medical Sciences and Public Health, University of Cagliari, Cagliari, Italy; 5 PhD Program in Capacities Building for Global Health, University of Cagliari, Cagliari, Italy; 6 Institute of Psychiatry, Federal University of Rio de Janeiro, Rio de Janeiro, RJ, Brazil

**Keywords:** anhedonia, chronic fatigue syndrome, depression, long COVID, mood disorders

## Abstract

**Background:**

Post-COVID chronic fatigue syndromes are frequently associated with depressive episodes and alterations in reward processing. Anhedonia, a core depressive symptom linked to reward-system dysfunction, may be particularly relevant in this context.

**Objective:**

To investigate whether depressive symptoms in individuals with post-COVID–like chronic fatigue syndrome display a distinctive configuration of core depressive features, particularly in the balance between depressed mood and anhedonia.

**Methods:**

In an exploratory cross-sectional comparative study with a matched historical control group, patients with post-COVID–like chronic fatigue syndrome were compared with matched controls from a pre-COVID community sample. Groups were matched for age, sex, and overall depression severity. Depressive symptoms were assessed using the Patient Health Questionnaire-9 (PHQ-9), and item-level scores were analyzed.

**Results:**

The post-COVID fatigue group and matched historical controls showed comparable overall depressive symptom severity and similar rates of mild and moderate depressive symptoms. However, depressed mood was significantly less pronounced in the post-COVID fatigue group, while anhedonia did not differ significantly between groups. Consequently, the depressed mood/anhedonia ratio was markedly reduced in the post-COVID fatigue group.

**Conclusions:**

Depressive symptoms in post-COVID–like chronic fatigue syndrome may show an altered balance between the two core depressive features assessed by the PHQ-9. In this exploratory comparison, the most robust finding was reduced depressed mood in the post-COVID fatigue group, whereas anhedonia was not significantly increased. These findings should therefore be interpreted as preliminary evidence of a different depressive symptom configuration, rather than as demonstration of an anhedonia-dominant phenotype.

## Introduction

Patients with post-COVID syndromes, in whom comorbidity with mood disorders is highly prevalent (1), exhibit marked slowed reaction times during cognitive tasks (2, 3). Deficits in cognitive responding have been associated with alterations in reward processing during task performance. Neuroimaging studies have demonstrated delayed activation of the left frontal gyrus and increased activity of the task-positive network during cognitive task execution (4). Altered reward/pleasure responsiveness may influence the syndromic expression of depression. In adolescents with myalgic encephalomyelitis/chronic fatigue syndrome (ME/CFS), a condition sharing several similarities with post-COVID fatigue, anhedonia has been found to be highly prevalent and, in most cases, associated with a diagnosis of depression (5). Likewise, in ME/CFS, similarly to what has been observed in post-COVID fatigue syndrome, multiple studies have reported alterations in reward/gratification systems (6–8). Anhedonia is defined as a marked reduction in the ability to experience pleasure or motivation toward previously rewarding activities. It represents one of the two core symptoms of major depressive episodes, together with depressed mood (9–11). Previous evidence indicates that anhedonia is common but not universal among individuals with major depressive disorder, supporting the view that the two core depressive symptoms may vary in their relative expression across patients (12–14). However, prevalence estimates of anhedonia in MDD cannot be directly translated into a fixed depressed mood/anhedonia ratio. In the present study, the depressed mood/anhedonia balance was therefore examined only as an exploratory within-study indicator of the relative intensity of the two PHQ-9 core depressive items. Although anhedonia has been linked in previous research to alterations in reward processing and motivational systems, the present study was not designed to directly investigate neurobiological mechanisms (13). Rather, it adopts a clinical and phenomenological approach, focusing on the relative expression of the two core depressive symptoms assessed by the PHQ-9: anhedonia and depressed mood (14). This approach may be particularly informative in post-COVID fatigue, a condition in which fatigue, reduced motivation, cognitive slowing, and affective symptoms frequently coexist and may partly overlap (13, 15). We therefore hypothesized that depressive symptoms in individuals with post-COVID–like chronic fatigue syndrome may show a distinctive configuration, characterized not necessarily by greater overall depression severity, but by a different balance between anhedonia and depressed mood. To test this hypothesis, we compared PHQ-9 item-level symptom profiles between individuals with post-COVID–like chronic fatigue syndrome and matched community controls, with matching performed for age, sex, and overall depressive symptom severity. The aim of the present study is to test this hypothesis by examining whether, in a sample of individuals with chronic fatigue syndrome and a very high frequency of depressive episodes, this distinctive symptom profile is observed (16).

## Methods

### Design

This was an exploratory cross-sectional comparative study with a matched historical control group. The analysis was conducted *post hoc* using baseline data from participants recruited for a parent randomized clinical trial registered at ClinicalTrials.gov (NCT05793736), designed to evaluate a biofeedback intervention in post-COVID fatigue syndrome. The present manuscript does not report the primary endpoint of that trial, but focuses on baseline depressive symptom configuration assessed with the PHQ-9. The clinical group consisted of individuals with post-COVID–like chronic fatigue syndrome assessed in 2023, whereas matched historical controls were selected from a pre-COVID regional community survey.

### Sample

The clinical group consisted of consecutive adult patients referred to the Psychiatric Liaison Unit of the University Hospital of Cagliari, Italy, from clinical units of two university hospitals of the University of Cagliari. Referral was based on the presence of persistent post-COVID fatigue symptoms requiring liaison psychiatric assessment within the context of a broader randomized clinical study, rather than on a primary diagnosis of major depression. Eligibility criteria for the parent study included age ≥18 years, a documented history of SARS-CoV-2 infection in the previous 12 months confirmed by PCR testing, an acute respiratory syndrome with fever, and subsequent fulfillment of the 2015 Institute of Medicine/National Academy of Medicine diagnostic criteria for ME/CFS in the post-COVID course. The main exclusion criterion was moderate-to-severe intellectual disability. Recruitment occurred at the University Hospital of Cagliari between February and May 2023. The present manuscript reports a secondary, exploratory analysis focused on depressive symptom configuration within this clinical sample. For the present secondary analysis, the available sociodemographic and clinical variables were age, sex, PHQ-9 total score, PHQ-9 item-level scores, PHQ-9 severity categories, and clinical eligibility for post-COVID–like chronic fatigue syndrome. No biological, neuroimaging, or structured diagnostic interview data were available for this specific analysis. The control group was drawn from a pre-COVID regional community survey conducted in Sardinia, Italy, which collected sociodemographic information and PHQ-9 data in the general population before the COVID-19 pandemic. Controls were selected from this database according to the matching procedure described below, using age, sex, and total PHQ-9 score as matching variables. Because the community survey was not designed as a medical study, participants did not undergo a clinical assessment for fatigue syndromes or post-infectious conditions. Therefore, the possibility that a small number of individuals with undetected fatigue syndromes were included among matched historical controls cannot be completely excluded. However, given the low estimated prevalence of ME/CFS in the general population, such contamination is likely to have been limited and would probably have biased results toward the null (17). Although eligibility required documented SARS-CoV-2 infection within the previous 12 months, individual-level information on the exact time elapsed between acute infection and psychiatric liaison assessment was not available in a sufficiently complete form for the present secondary analysis. Reliable and comparable information on current psychopharmacological treatment was not available for both groups in the present secondary analysis. Therefore, medication exposure could not be included as a covariate or examined as a potential modifier of depressive symptom configuration. Recruitment at the University Hospital of Cagliari, Italy, occurred from February to May 2023.

### Study tool

Demographic information was collected using a structured instrument already used in similar studies. The self-administered Patient Health Questionnaire-9 (PHQ-9) (18, 19) in the Italian version (20, 21) was adopted to identify the presence of depressive symptoms in the last 2 weeks. The PHQ-9 items correspond to the nine “core criteria” symptoms for a diagnosis of a depressive episode according to the most widely used psychiatric classification, the DSM-5 (22). The degree of severity is measured for each symptom on a from 0 (corresponding to “not at all”) to 3 (“maximum severity or presence every day”). The cut-off of 4/5 identifies a depressive episode of at least mild severity; a cut-off of 9/10 a depressive episode of at least moderate severity (18).

### Statistical analysis

PHQ-9 total scores and item-level scores were summarized using means and standard deviations to describe overall depressive symptom severity and the relative intensity of individual symptoms. Categorical variables were summarized as frequencies and percentages. Between-group comparisons of mean scores were performed as exploratory analyses using one-way ANOVA, while categorical variables were compared using chi-square tests with Yates’ correction when appropriate. For each participant in the post-COVID fatigue group, a pool of eligible matched historical controls was created from the community survey database. Controls were required to have the same sex, a similar age (± 2 years), and a similar total PHQ-9 score (± 2 points). In addition, matching was constrained so that the PHQ-9 severity category was preserved with respect to the cutoff for moderate depressive symptoms: participants with PHQ-9 scores >9 were matched only with controls who also had PHQ-9 scores >9, whereas participants with PHQ-9 scores ≤9 were matched only with controls who had PHQ-9 scores ≤9. Two controls were then selected for each participant without replacement. Because PHQ-9 item scores are four-point ordinal variables and the sample size was small, item-level comparisons should be interpreted as exploratory comparisons of mean symptom intensity rather than as confirmatory parametric tests. Individual-level item distributions were not available in a sufficiently complete form to allow reliable testing of normality, homogeneity of variance, non-parametric reanalysis, or 4×2 comparisons of PHQ-9 response categories. Standardized effect sizes were calculated using Hedges’ g to estimate the magnitude of between-group differences. To reduce the risk of false-positive findings due to multiple testing, a Bonferroni correction was applied to the nine PHQ-9 item-level comparisons, resulting in a corrected significance threshold of p < 0.0056. Findings not surviving correction were interpreted as non-significant descriptive differences. The depressed mood/anhedonia ratio was treated as an *a priori*, exploratory descriptive index of the relative intensity of the two PHQ-9 items corresponding to the two DSM core depressive symptoms (17, 23). This ratio was not selected from an exploratory comparison of all possible PHQ-9 item ratios. It should not be interpreted as a prevalence ratio, diagnostic criterion, validated reference ratio for major depressive disorder, external benchmark, phenotype-defining measure, or independent confirmatory endpoint. The PHQ-9 item assessing “little interest or pleasure in doing things” was used as a brief screening indicator of anhedonic symptom intensity and should not be considered equivalent to a dedicated anhedonia scale such as the SHAPS (23).

### Ethics

The study was conducted according with the principles of the Helsinki Declaration (24, 25). A schedule for informed consent was signed by each participant before the interview. The institutional ethics committee of the University Hospital of Cagliari, Italy, approved the study, protocol number NP/2023/496. The study is also registered on ClinicalTrials.gov under ID NCT05793736. Recruitment at the University Hospital of Cagliari, Italy, occurred from February to May 2023. The study is funded by the Fondazione Banco di Sardegna and PNRR - PE13 INF-ACT One Health Basic and Translational Research Actions addressing Unmet Needs on Emerging Infectious Diseases PE00000007.

## Results

Our findings should be considered exploratory and hypothesis-generating. **Table 1** shows the characteristics of the study samples. The frequency of women (88.2%), the frequency of people with a PHQ score >4, depressive episode of at least mild severity (88.2%), and PHQ score >9, depressive episode of at least moderate severity (58.8%) are comparable between the two groups. A small and identical proportion of participants in both groups scored below the PHQ-9 cutoff for at least mild depressive symptoms (2/17 in the post-COVID fatigue group and 4/34 among matched historical controls). These participants were retained to preserve the original matched comparison across the full PHQ-9 severity range. Therefore, the analysis should be interpreted as an exploratory comparison of depressive symptom configuration rather than as a study restricted to participants with clinically relevant depressive episodes. The mean age (49.52 ± 11.91 vs. 49.08 ± 11.68) and the mean PHQ-9 total score (10.23 ± 5.25 vs. 9.35 ± 4.52) do not show statistically significant differences due to the matching techniques. The main statistically supported item-level finding was reduced depressed mood in the post-COVID fatigue group. The altered depressed mood/anhedonia ratio should be interpreted as reflecting a different balance between the two core depressive symptoms, mainly driven by reduced depressed mood, rather than as evidence of a statistically confirmed increase in anhedonia. As expected from the matching procedure, the proportion of participants with PHQ-9 scores >9 was identical in the two groups.

**Table 1:** Study samples.

Item	Post-COVID like fatigue syndrome N=17	Controls N=34	F	Statistics
Sex (females)	15 (88.2%)	30 (88.2%)	Matched	Chi-square 1df =0 p=1
Mean age	49.52 ± 11.91	49.08 ± 11.68	Matched	ANOVA 1,49 df F = 0.016 p=0.900
Total PHQ-9 score	10.23 ± 5.25	9.35 ± 4.52	Matched	ANOVA 1,49 df F = 0.303 p=0.585
PHQ score >4 depressive episode of at least mild severity	15 (88.2%)	30 (88.2%)	Matched	Chi-square 1df =0 p=1
PHQ score >9 depressive episode of at least moderate severity	10 (58.8%)	20 (58.8%)	Matched	Chi-square 1df =0 p=1

**Table 2** reports the mean scores for each of the nine PHQ-9 items in the two groups. After correction for multiple comparisons, the only PHQ-9 item-level difference that remained statistically significant was depressed mood (PHQ-9 item 2), which was lower in the post-COVID fatigue group than in controls (0.58 ± 0.76 vs. 1.55 ± 0.88; p < 0.0001; Hedges’ g = −1.13). Anhedonia showed only a small, non-significant difference in mean symptom intensity in the post-COVID fatigue group compared with controls (PHQ-9 item 1: 1.29 ± 0.82 vs. 1.11 ± 0.93; p = 0.502; Hedges’ g = 0.20). The present findings should not be interpreted as demonstrating a higher prevalence of anhedonia in post-COVID fatigue. Rather, anhedonia showed a small and non-significant difference in mean intensity, whereas depressed mood was significantly lower in the post-COVID fatigue group. Therefore, the altered depressed mood/anhedonia balance appears to be mainly driven by reduced depressed mood rather than by a statistically confirmed increase in anhedonia. The depressed mood/anhedonia ratio was markedly lower in the post-COVID fatigue group than in controls (0.44 ± 0.92 vs. 1.39 ± 0.94; p = 0.002; Hedges’ g = −1.00). This ratio is reported only to describe the relative balance between the two PHQ-9 core depressive items. The primary item-level finding remains the significantly lower score for depressed mood, whereas anhedonia did not differ significantly between groups. No other PHQ-9 item-level comparison survived correction for multiple comparisons; therefore, differences in fatigue/low energy, concentration difficulties, and suicidal ideation should be interpreted as non-significant descriptive differences only. Because PHQ-9 item scores are ordinal variables, the item-level comparisons reported in **Table 2** should be interpreted as exploratory comparisons of mean symptom intensity. After Bonferroni correction for the nine PHQ-9 item-level comparisons, depressed mood remained the only item showing a statistically significant between-group difference.

**Table 2:** Mean score PHQ-9 in post-COVID like fatigue syndromes and controls.

Item	Post-COVID like fatigue syndrome	Controls	ANOVA 1,49 df	p
PHQ1 Little interest or pleasure in doing things	1.29 ± 0.82	1.11 ± 0.93	F=0.458	p=0.502
PHQ2 Feeling down, depressed or hopeless	0.58± 0.76	1.55 ± 0.88	F=15.327	p<0.0001
PHQ3 Trouble falling asleep, staying asleep, or sleeping too much	1.35 ± 0.76	1.47 ± 1.14	F=0.153	p=0.697
PHQ4 Feeling tired or having little energy	2.00 ± 0.76	1.55 ± 0.88	F=3.232	p=0.078
PHQ5 Poor appetite or overeating	1.11 ± 0.86	0.85 ± 1.06	F=0.768	p=0.385
PHQ6 Feeling bad about yourself - or that you’re a failure or have let yourself or your family down	0.94 ± 0.99	0.91 ± 0.85	F=0.013	p=0.921
PHQ7 Trouble concentrating on things, such as reading the newspaper or watching television	1.35 ± 0.90	0.94 ± 0.93	F=2.249	p=0.140
PHQ8 Moving or speaking so slowly that other people could have noticed. Or, the opposite - being so fidgety or restless that you have been moving around a lot more than usual	0.82 ± 0.70	0.85 ± 1.08	F=0.011	p=0.918
PHQ-9 Thoughts that you would be better off dead or of hurting yourself in some way	0.35 ± 0.47	0.20 ± 0.40	F=1.269	p=0.266

## Discussion

The present study investigated whether depressive symptoms occurring in individuals with post-COVID–like chronic fatigue syndrome show an altered configuration of core depressive features, with particular reference to the relative intensity of depressed mood and anhedonia. The main statistically supported finding of this exploratory study was a lower representation of depressed mood in the post-COVID fatigue group, despite comparable overall PHQ-9 severity between groups. Anhedonia showed only a small and non-significant difference in the opposite direction. Therefore, the altered depressed mood/anhedonia ratio should be interpreted as reflecting a different balance between the two core depressive symptoms, mainly driven by reduced depressed mood, rather than as evidence of a confirmed increase in anhedonia. This interpretation is also consistent with the broader recognition that depressive syndromes are clinically heterogeneous and may be composed of multiple symptom combinations. As emphasized by Østergaard et al., the same categorical diagnosis of major depression may arise from markedly different constellations of symptoms. In this context, the present findings should not be read as identifying a new depressive subtype, but rather as an exploratory observation that one core depressive item, depressed mood, was less represented in the post-COVID fatigue group despite comparable overall PHQ-9 severity (26).

This finding is clinically relevant because it suggests that depressive symptom expression in post-COVID fatigue may not simply mirror conventional depressive presentations observed in the general population. However, the present data do not demonstrate a distinct anhedonia-dominant phenotype. Rather, they provide an exploratory phenomenological signal that requires replication in larger, clinically characterized samples. These findings provide preliminary and hypothesis-generating support for an altered balance between core depressive symptoms in post-COVID fatigue, primarily driven by reduced depressed mood rather than by increased anhedonia (12, 27, 28). Because psychopharmacological treatment data were not available in a comparable form across groups, the observed symptom pattern should not be interpreted as independent of medication exposure. Given the ordinal nature of PHQ-9 item responses and the small sample size, these item-level findings should be interpreted cautiously. The principal observation is not a definitive categorical difference in depressive subtypes, but an exploratory signal suggesting an altered balance between depressed mood and anhedonia in the post-COVID fatigue group. Despite the clinical group and the matched historical control group being closely matched for age, sex, and overall severity of depressive symptoms, as reflected by comparable total PHQ-9 scores and identical proportions of mild and moderate depressive episodes, the relative expression of selected core depressive symptoms differed between groups in an exploratory analysis. Specifically, depressed mood (PHQ-9 item 2) was significantly less pronounced in individuals with post-COVID fatigue compared with community controls, whereas anhedonia (PHQ-9 item 1) showed a small non-significant difference in the post-COVID fatigue group. As a consequence, the ratio between depressed mood and anhedonia was markedly altered in the post-COVID fatigue group (0.4) compared with matched historical controls (1.4). This finding should not be interpreted as divergence from an established depressed mood/anhedonia ratio in major depressive disorder. Rather, it indicates that, within the present matched comparison, depressed mood was represented less strongly relative to anhedonia in the post-COVID fatigue group than in the matched historical control group. Importantly, after correction for multiple comparisons, the only PHQ-9 item-level difference that remained statistically significant was the lower score for depressed mood in the post-COVID fatigue group. Therefore, the present findings should be interpreted as evidence of an altered balance between depressed mood and anhedonia, mainly driven by reduced depressed mood rather than by a statistically confirmed increase in anhedonia. This finding should be interpreted primarily as a clinical phenomenological observation. The present data do not demonstrate a specific neurobiological mechanism; rather, they indicate that, at comparable levels of overall depressive symptom severity, individuals with post-COVID–like fatigue may express depression through a different balance of core symptoms. In particular, the lower prominence of depressed mood suggests a descriptive alteration in the relative intensity of depressed mood and anhedonia, driven principally by lower depressed mood. This interpretation is consistent with, but does not directly prove, previous models linking chronic fatigue states, post-infectious syndromes, and depressive anhedonia to altered reward processing, effort-based decision-making, and fronto-striatal functioning. Accordingly, the neurobiological literature should be viewed as a framework for generating hypotheses rather than as evidence directly tested in the present study. Future studies integrating clinical assessment with inflammatory, neurocognitive, and neuroimaging measures will be necessary to determine whether the altered symptom balance observed here corresponds to distinct biological pathways (23, 29–31). Such a profile is consistent with neurobiological models linking fatigue syndromes to dysfunctions in reward processing and fronto-striatal circuits, as documented in both post-COVID conditions and ME/CFS (6–8). Neuroimaging studies in these populations have demonstrated altered activation of basal ganglia and frontal regions during tasks requiring effort, reward anticipation, or cognitive engagement, mechanisms that are closely related to anhedonic symptomatology rather than to depressed mood per se (32–35). The absence of significant group differences in most of the remaining PHQ-9 items further supports the specificity of this finding. Symptoms such as sleep disturbance, appetite changes, psychomotor alterations, and negative self-evaluation were comparable between groups, suggesting that the depressive syndromes observed in post-COVID fatigue do not differ globally from those seen in the general population, but rather diverge in their core affective–motivational components (26, 36–38). Notably, there were non-significant numerical differences, with higher scores in the post-COVID fatigue group for fatigue/low energy (item 4) and concentration difficulties (item 7), symptoms that overlap with the defining features of chronic fatigue syndromes. Although these differences did not reach statistical significance, they point toward a possible amplification of cognitive–energetic symptoms within depressive episodes occurring in this clinical context (39). The absence of a statistically significant difference in PHQ-9 item 4 (“feeling tired or having little energy”) should also be interpreted cautiously. This item is non-specific and captures fatigue as a depressive symptom over the previous two weeks, rather than the broader clinical construct of post-COVID fatigue syndrome. Moreover, the matched historical control group had comparable overall depressive symptom severity, which may partly explain similar scores on fatigue-related depressive items. The neurobiological literature on reward processing, motivational impairment, inflammation, and post-infectious fatigue provides a plausible conceptual framework for interpreting these findings, but the present study did not directly assess biological, inflammatory, neurocognitive, or neuroimaging markers. Therefore, any mechanistic interpretation should be considered hypothetical and hypothesis-generating.

Taken together, these results align with previous evidence indicating that anhedonia is moderately prevalent in conditions characterized by chronic fatigue and reduced reward sensitivity, such as ME/CFS, and that depressive states with prominent anhedonia may represent a neurobiologically distinct altered depressive symptom configuration. Importantly, anhedonia has been associated with poorer treatment response to standard antidepressant therapies and with greater functional impairment (14). If depressive episodes in post-COVID fatigue are indeed characterized by a reconfiguration of core depressive symptom expression, with reduced depressed mood as the principal contributing feature, this may help explain the frequently reported suboptimal response to conventional treatments and highlights the need for tailored therapeutic approaches targeting motivational and reward-processing dysfunctions (40, 41). It should be emphasized, however, that a consistent literature indicates that anhedonia symptoms are markedly more frequent in bipolar spectrum disorders than in MDD (42–45). In conditions of strong immune activation, such as celiac disease (46) and hepatitis C (47), a high frequency of specific syndromes such as Recurrent Brief Depression has also been reported, a little-studied syndrome, although of recognized importance today, considered part of the bipolar spectrum (48). These considerations suggest particular attention to the syndromic typifications of mood disorders in conjunction with fatigue syndromes and more generally with syndromes with chronic immune activation because a better understanding and characterization of the clinical pictures can have important consequences in the setting of treatment and prognosis (49–53). This dimensional approach allows for a more refined understanding of depressive phenomenology in post-COVID conditions (54–56).

The distinction between depressed mood and anhedonia is clinically relevant because these symptoms may reflect partly different dimensions of depressive psychopathology. While depressed mood is more closely related to negative affective experience, anhedonia involves reduced pleasure, motivation, and reward responsiveness. In the present study, the altered balance between these two symptoms may therefore indicate a clinically meaningful shift in depressive phenomenology among individuals with post-COVID–like fatigue. However, because no biological or neuroimaging measures were collected, any interpretation in terms of dopaminergic, inflammatory, or fronto-striatal mechanisms remains speculative and should be tested in future studies (57, 58). In contrast, anhedonia has been more specifically linked to abnormalities in mesolimbic dopaminergic pathways, particularly involving the ventral striatum and nucleus accumbens, and to deficits in reward anticipation, motivation, and reinforcement learning (13, 59, 60).

This neurobiological dissociation has been further supported by neuroimaging studies showing differential patterns of brain activation in patients with predominant anhedonia compared to those with more pronounced affective distress, as well as by pharmacological data indicating that conventional serotonergic antidepressants may preferentially improve depressed mood, while dopaminergic or glutamatergic interventions may be more effective in targeting anhedonic symptoms (15, 61). Within this framework, the altered depressed mood/anhedonia ratio observed in the present study may reflect a relative predominance of reward-system dysfunction over stress-related affective dysregulation in post-COVID fatigue syndromes (52, 62). This interpretation is consistent with the growing literature describing post-infectious and inflammatory conditions as being characterized by motivational deficits, reduced reward sensitivity, and altered effort-based decision-making, possibly mediated by inflammatory effects on dopaminergic signaling (29). Altogether, these findings support the hypothesis that depressive presentations in post-COVID fatigue may involve a shift in core depressive symptom expression, primarily reflecting reduced depressed mood rather than increased anhedonia. This interpretation remains exploratory and should be tested in prospective studies. These findings should be interpreted in light of the methodological limitations described below.

## Limitations

Several limitations should be considered when interpreting the present findings. First, the sample size was small, limiting statistical power and the stability of effect-size estimates. Second, depressive symptoms were assessed using the PHQ-9, a self-report screening instrument, rather than a structured clinical interview. Therefore, the findings should be interpreted as referring to PHQ-9 depressive symptom configuration rather than to clinically diagnosed depressive episodes or depressive subtypes. In addition, the sample was not restricted to participants above the PHQ-9 cutoff for clinically relevant depressive symptoms, although the proportion of participants below this threshold was small and identical in both groups. Third, the study design was an exploratory cross-sectional comparison with a matched historical control group. The post-COVID fatigue group was derived from a liaison psychiatry referral pathway, whereas controls were selected from a pre-COVID community survey. Although matching reduced differences in age, sex, and overall depressive symptom severity, residual bias related to recruitment context, period effects, medical burden, help-seeking behavior, and unmeasured clinical characteristics cannot be excluded. The community survey was not designed as a medical study, and participants did not undergo clinical screening for fatigue syndromes or post-infectious conditions. Any undetected fatigue condition among matched historical controls would probably have biased results toward the null, but this possibility cannot be completely excluded. Fourth, several clinically relevant variables were unavailable for this secondary analysis. Reliable and comparable data on current psychopharmacological treatment were not available for both groups, although medication exposure may influence anhedonia, depressed mood, energy, sleep, and cognitive symptoms. Individual-level information on the exact time elapsed between acute SARS-CoV-2 infection and psychiatric liaison assessment was also unavailable, preventing analysis of whether depressive symptom configuration varied according to illness duration. Finally, the study lacked biological, inflammatory, neurocognitive, and neuroimaging measures, precluding direct testing of the hypothesized neurobiological mechanisms. Fifth, individual PHQ-9 items are four-point ordinal measures. Because this secondary analysis was based on the available summarized dataset, individual-level item distributions were not available in a sufficiently complete form to allow reliable testing of normality, homogeneity of variance, non-parametric reanalysis, or 4×2 comparisons of PHQ-9 item response categories. Therefore, item-level comparisons should be interpreted as exploratory comparisons of mean symptom intensity. Although effect sizes were reported and Bonferroni correction was applied, the statistical findings remain preliminary. A further limitation concerns the assessment of anhedonia and the novelty of the depressed mood/anhedonia ratio. PHQ-9 item 1 is a brief screening item assessing reduced interest or pleasure over the previous two weeks and cannot be considered equivalent to dedicated anhedonia instruments such as the SHAPS. The depressed mood/anhedonia ratio is a novel, non-validated, exploratory index of relative PHQ-9 item intensity. Although it was conceptually restricted to the two DSM core depressive symptoms, many alternative ratios between PHQ-9 items could theoretically be constructed. Therefore, this ratio should be interpreted only as a descriptive hypothesis-generating measure and not as evidence establishing a distinct depressive phenotype. Finally, the present analysis was not the primary registered endpoint of the parent randomized clinical trial, which was designed to evaluate a biofeedback intervention in post-COVID fatigue syndrome. The depressive symptom analysis reported here was conducted *post hoc* using baseline data. Prospective studies specifically designed to examine depressed mood and anhedonia in post-COVID fatigue should include structured diagnostic interviews, dedicated anhedonia measures such as the SHAPS, longitudinal follow-up, medication assessment, and analytic approaches suited to ordinal symptom data.

## Conclusion

The present exploratory findings suggest that depressive symptoms in individuals with post-COVID–like chronic fatigue syndrome may show a different configuration of core depressive features compared with matched historical controls. The most robust finding was reduced depressed mood despite comparable overall depressive symptom severity. Anhedonia was not significantly increased in the post-COVID fatigue group and should therefore not be interpreted as the defining feature of a distinct depressive phenotype. Rather, the observed depressed mood/anhedonia ratio may indicate an altered internal balance of depressive symptoms, primarily driven by lower depressed mood. These results should be considered preliminary and hypothesis-generating. Replication in larger samples, with individual-level longitudinal data, structured clinical interviews, medication assessment, dedicated anhedonia measures, and analytic methods suited to ordinal symptom data, is required before drawing conclusions about specific depressive phenotypes or underlying neurobiological mechanisms in post-COVID fatigue.

## Data Availability

The raw data supporting the conclusions of this article will be made available by the authors, without undue reservation.
